# Supplementary material for: Can secondary contact following range expansion be distinguished from barriers to gene flow?
Source: PeerJ. 2018 Oct 1;6:e5325. doi: 10.7717/peerj.5325 (PMC6171497; doi:10.7717/peerj.5325)
Supplement: Supplemental Information 1 [file peerj-06-5325-s001.pdf]

Can secondary contact following range expansion be distinguished from barriers to gene flow? – Supplementary Information

Johanna Bertl[1,*] Harald Ringbauer[2,3]
Michael G. B. Blum[4]

June 20, 2018

[1] Department of Molecular Medicine (MOMA), Aarhus University, Aarhus, Denmark
[2] Institute of Science and Technology Austria, Klosterneuburg, Austria
[3] Department of Human Genetics, University of Chicago, Chicago, United States
[4] Univ. Grenoble Alpes, CNRS, Laboratoire TIMC-IMAG, Université Grenoble Alpes, CNRS, Grenoble, France
* *corresponding author*

S1 Examples of summary statistics along the range of demes

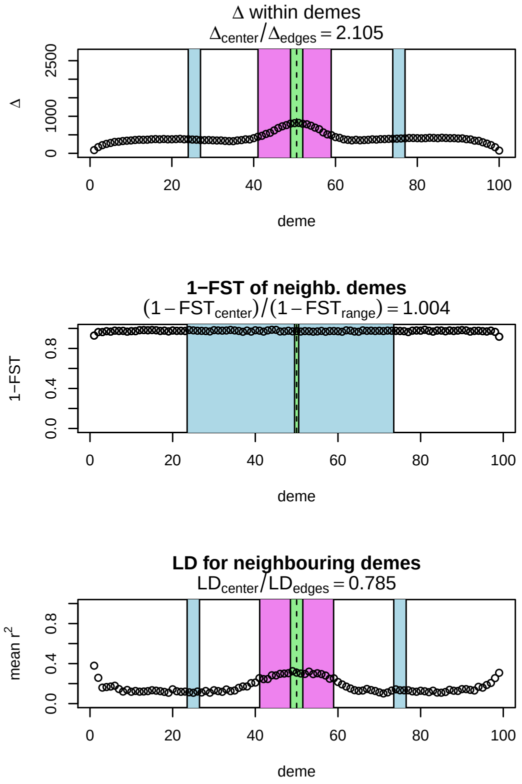

Figure S1: Simulation results for 1 replicate of the secondary contact model with $t_C = 1$. For Δ and LD, the subscript *center* denotes the mean over demes 49–52 (green zone) and *edges* over demes 24–27 and 74–77 (blue zone). For F_{ST}, *center* denotes demes 50 and 51 (green zone) and *range* the mean over the neighboring demes in 26–74 except demes 50 and 51 (blue zone). Pink: admixture zone; width $2\sigma\sqrt{t_C} = 8.94$ in either direction from the center of the zone.

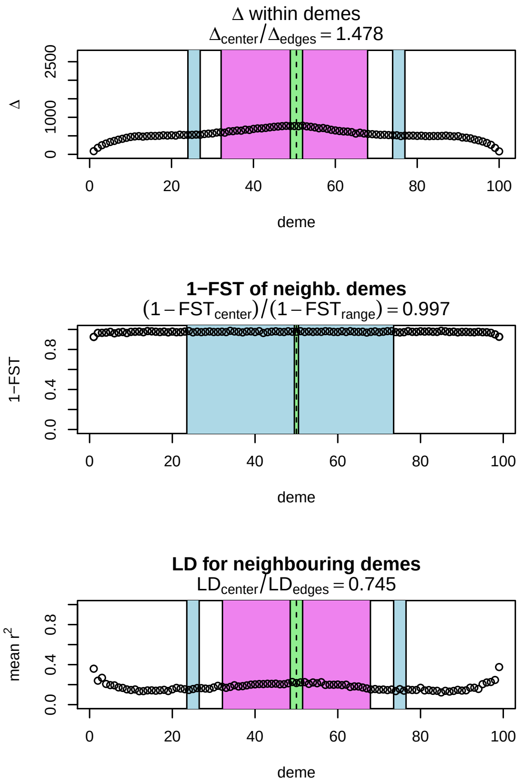

Figure S2: Simulation results for 1 replicate of the secondary contact model with $t_C = 4$. See fig. S1 for a description. Admixture zone width $2\sigma\sqrt{t_C} = 17.9$ in either direction from the center of the zone.

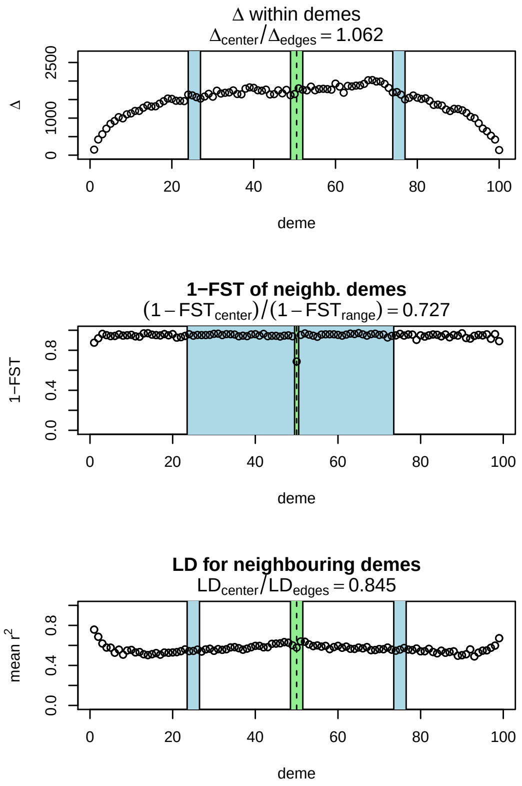

Figure S3: Simulation results for 1 replicate of the stepping stone model with $m_B = 2$. For Δ and LD, the subscript *center* denotes the mean over demes 49–52 (green zone) and *edges* over demes 24–27 and 74–77 (blue zone). For the F_{ST}, *center* denotes demes 50 and 51 (green zone) and *range* the mean over the neighboring demes in 26–74 except demes 50 and 51 (blue zone).

S2 Comparison with expectations from the diffusion theory

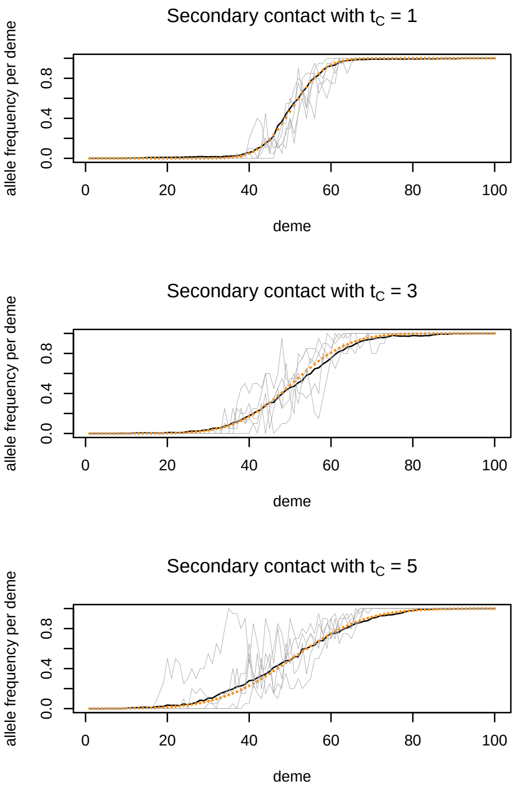

Figure S4: Comparison between observed and predicted allele frequency clines in the secondary contact model. Clinal SNPs with minor allele frequency below 0.05 in demes 1-10 and above 0.95 in demes 91-100 were selected. Gray lines: simulation results for 5 individual unlinked SNPs. Black line: mean over 100 unlinked SNPs. Orange dotted line: Predicted allele frequency cline from Sedghifar et al. (2015); cumulative normal distribution with mean 50.5 and variance $\sigma^2 t_C$.

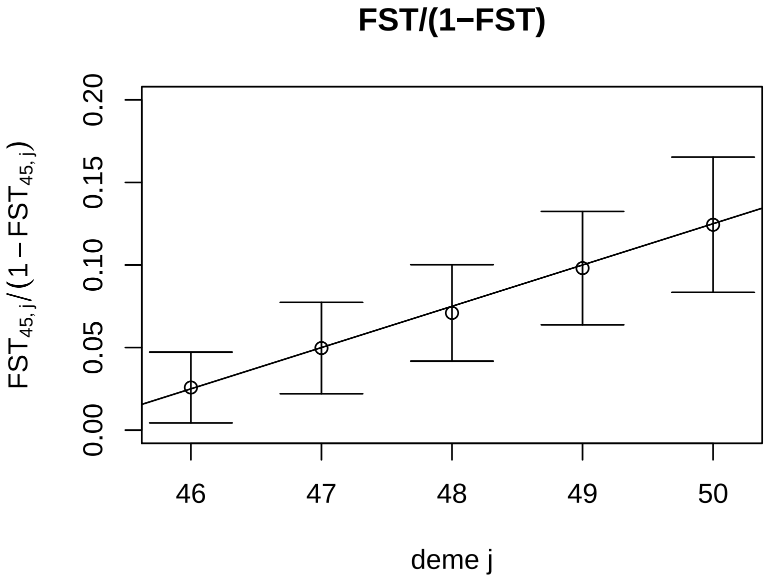

Figure S5: Comparison of the simulation results of the stepping stone model with no barrier ($m_B/m = 1$) with theory predictions. Rousset (1997) showed that for a one dimensional habitat pairwise $F_{ST}/(1 - F_{ST})$ increases linearly with geographic distance r for pairs of subpopulations with slope $1/(4N\sigma^2)$, as long as $\sqrt{2mr}/\sigma \ll 1$. Here, we compare this theoretical prediction to our simulation results. Dots: Mean over 100 replicates. Error bars: ± 2 standard deviations over 100 replicates. Straight lines depict theory predictions, with slope 1/20 ($\sigma^2 = 2m = 20$, $4N = 1$).

S3 Simulations at additional parameter combinations

To assess the sensitivity of the results with regard to the other parameters of the models, we resimulated under the secondary contact model with $t_C = 1$ and varied the bottleneck intensity (figure S6) and the expansion speed (d; figure S7). For comparison, the stepping stone model is shown with the same parameters as in the main text.

In addition, we vary the migration rate m simultaneously in both models, in the secondary contact model with $t_C = 1$ and in the stepping stone model with $m_B/m = 0.002$ (figure S8).

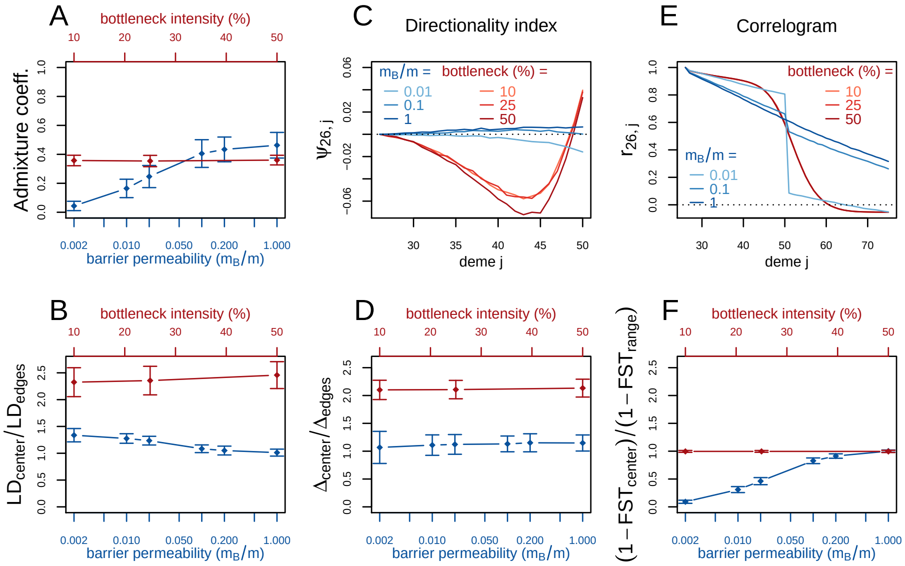

Figure S6: Red (lines and axis): secondary contact model with $t_C = 1$ and varying bottleneck intensity. Blue (lines and axis): stepping stone model with barrier. The dots denote the mean and the error bars ± 2 standard errors, estimated from 100 replicates of the simulations. The admixture coefficient is computed for the 5 demes to the left of the contact zone, demes 46–50. For Δ and LD, the subscript *center* denotes the mean over demes 49–52 and *edges* over demes 24–27 and 74–77. For the F_{ST}, *center* denotes demes 50 and 51 and *range* the mean over the neighboring demes in 26–74 except demes 50 and 51. (For these statistics, the edges of the range are dismissed because of the edge-effect in the stepping-stone model.) For the allele frequency correlogram and the ψ statistic, only the mean is plotted. Note that the correlogram is so similar for each of the three parameters that they are plotted on top of each other.

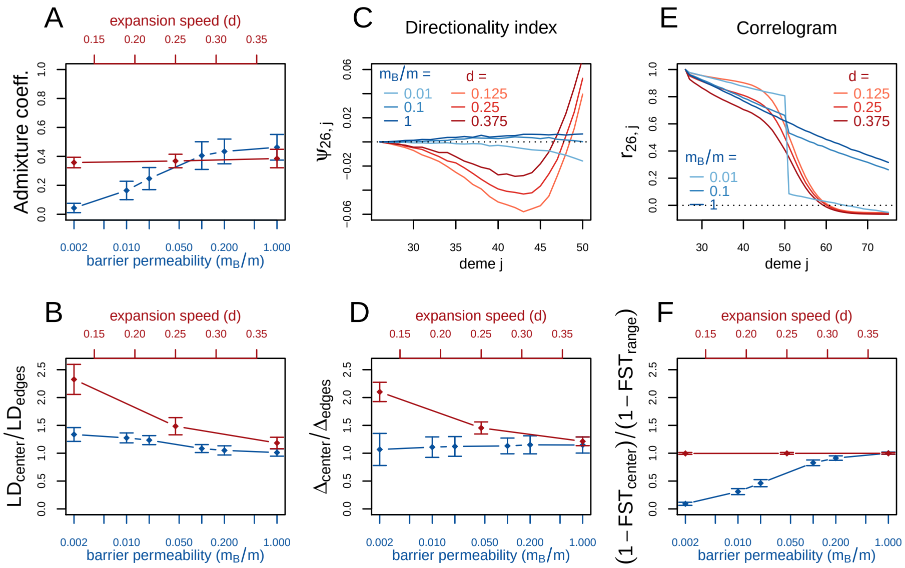

Figure S7: Red (lines and axis): secondary contact model with $t_C = 1$ and varying expansion speed (d). Blue (lines and axis): stepping stone model with barrier. Definitions of the summary statistics as in fig. S6.

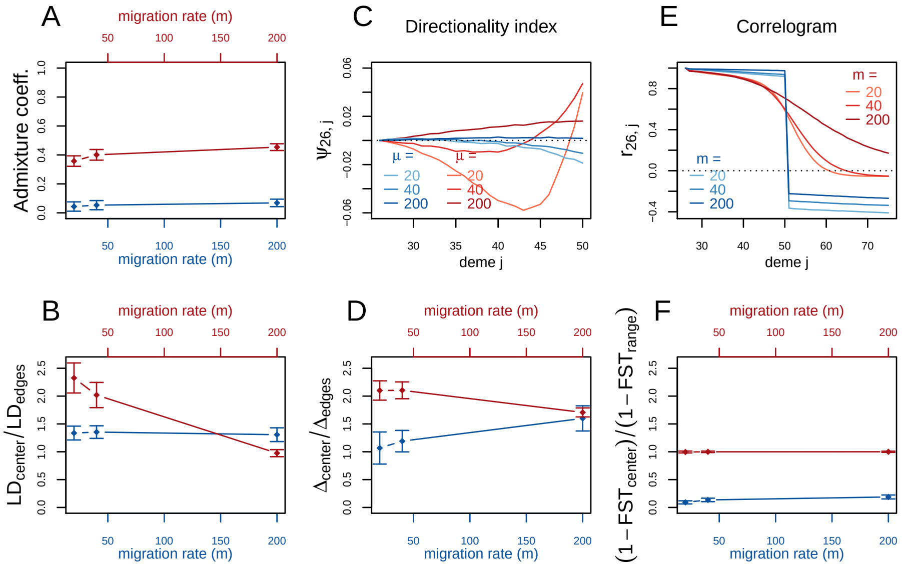

Figure S8: Red (lines and axis): secondary contact model with $t_C = 1$ and varying migration rate (m). Blue (lines and axis): stepping stone model with varying migration rate(m). The barrier permeability $m_B/m = 0.002$ is constant. Definitions of the summary statistics as in fig. S6.

S4 Different sampling schemes

We repeat the analysis with smaller datasets. Instead of 100 independent loci, we subsample the data and compute the summary statistics on 10 or 20 loci, respectively (fig. S9 and S10. We also simulate new data with only 4 genomes per deme (fig. S11 instead of 20). In addition, we compute the summary statistics for a dataset, where 20 genomes have been sampled from every 5th deme only (deme $5, 10, \ldots, 50, 51, 56, \ldots, 96$), so the data consists of DNA sequences from 20 demes (fig. S12).

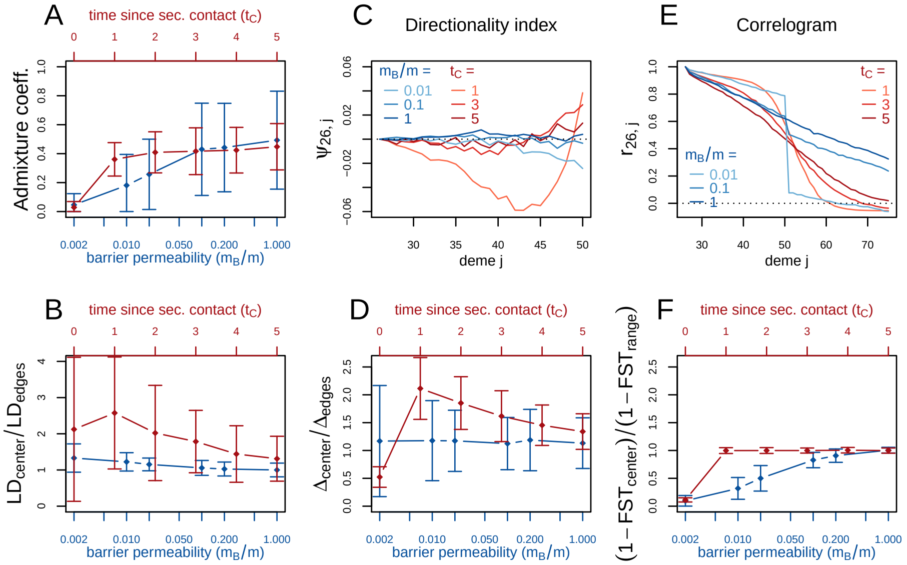

Figure S9: Smaller dataset: 10 independent loci. Red (lines and axes): secondary contact model; blue: stepping-stone model; both models as in fig. 3. Definitions of the summary statistics as in fig. S6. Note the rescaled y-axis in the bottom left panel (LD).

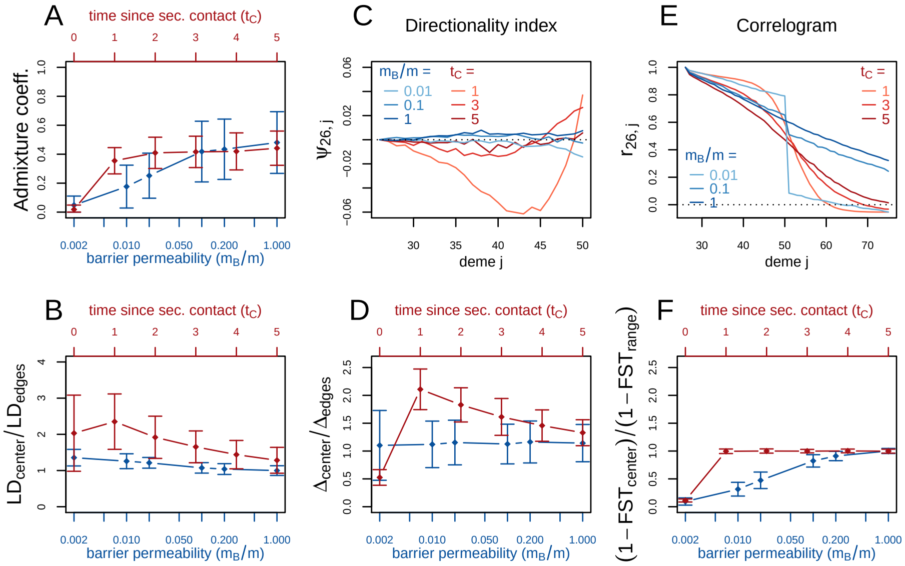

Figure S10: Smaller dataset: 20 independent loci. Red (lines and axes): secondary contact model; blue: stepping-stone model; both models as in fig. 3. Definitions of the summary statistics as in fig. S6. Note the rescaled y-axis in the bottom left panel (LD).

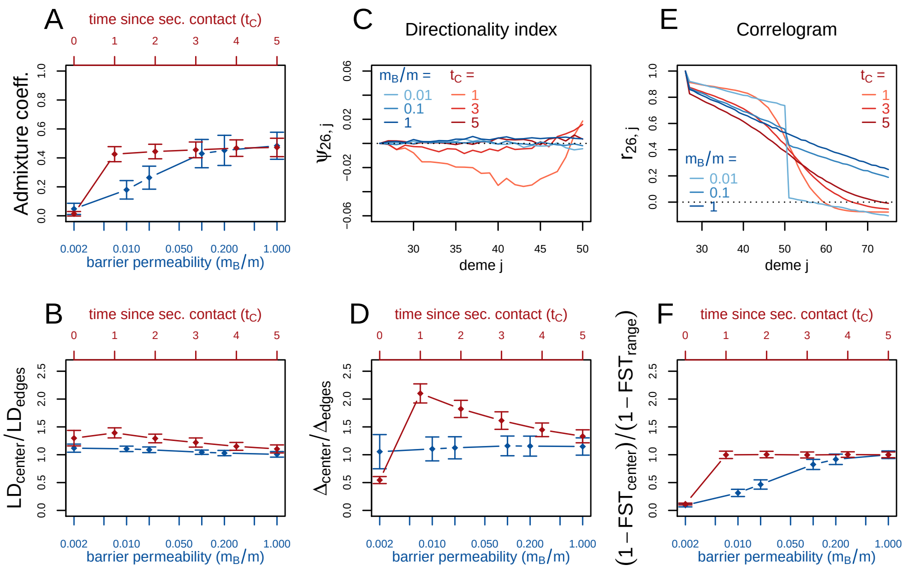

Figure S11: Smaller dataset: 4 genomes per deme. Red (lines and axes): secondary contact model; blue: stepping-stone model; both models as in fig. 3 (main text). Definitions of the summary statistics as in fig. S6.

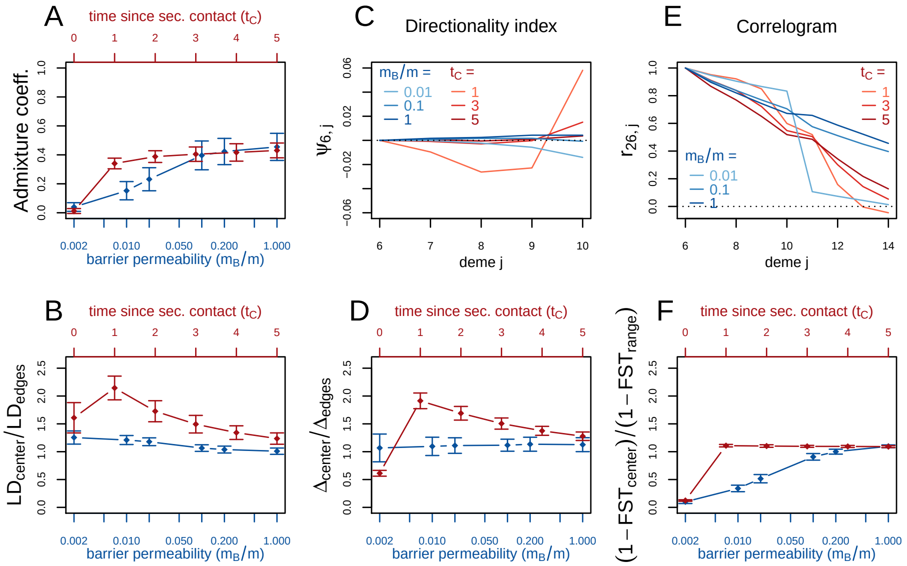

Figure S12: Smaller dataset: 20 demes. Red (lines and axes): secondary contact model; blue: stepping-stone model; both models as in fig. 3. Definitions of the summary statistics as in fig. S6.

S5 Tables of the results

This section contains the underlying values for the admixture coefficient, $LD_{\text{center}}/LD_{edges}$, $\Delta_{\text{center}}/\Delta_{\text{edges}}$ and $(1 - FST_{\text{center}})/(1 - FST_{range})$ of figures 3 and 4.

The tables S1–S4 have the following format: Left: Secondary contact model with different times since secondary contact (t_C); middle: secondary contact model with a moderate barrier to gene flow ($m_B/m = 0.1$) at the secondary contact zone; right: stepping-stone model with barrier to gene flow of different intensities (m_B/m; $m = 20$). The mean and standard error (se) of the admixture coefficient over 100 replicates of the simulations.

Secondary contact			with barrier		Stepping stone		
t_C	mean	se	mean	se	m_B/m	mean	se
0	0.011	0.008	0.011	0.008	0.002	0.044	0.016
1	0.358	0.018	0.187	0.018	0.010	0.165	0.032
2	0.403	0.020	0.259	0.021	0.020	0.247	0.038
3	0.418	0.026	0.298	0.023	0.100	0.405	0.048
4	0.428	0.031	0.320	0.026	0.200	0.434	0.043
5	0.442	0.027	0.339	0.029	1.000	0.463	0.044

Table S1: Admixture coefficient.

Secondary contact			with barrier		Stepping stone		
t_C	mean	se	mean	se	m_B/m	mean	se
0	1.886	0.187	1.886	0.187	0.002	1.337	0.062
1	2.326	0.135	2.435	0.118	0.010	1.275	0.045
2	1.863	0.110	2.094	0.133	0.020	1.235	0.041
3	1.601	0.087	1.856	0.091	0.100	1.084	0.036
4	1.421	0.073	1.636	0.076	0.200	1.050	0.041
5	1.288	0.062	1.510	0.078	1.000	1.012	0.032

Table S2: LD ratio.

Secondary contact			with barrier		Stepping stone		
t_C	mean	se	mean	se	m_B/m	mean	se
0	0.540	0.027	0.540	0.027	0.002	1.067	0.144
1	2.101	0.087	1.685	0.074	0.010	1.109	0.092
2	1.827	0.079	1.609	0.073	0.020	1.122	0.089
3	1.612	0.063	1.490	0.051	0.100	1.131	0.071
4	1.450	0.051	1.362	0.056	0.200	1.151	0.081
5	1.331	0.052	1.271	0.053	1.000	1.147	0.072

Table S3: Δ ratio.

Secondary contact			with barrier		Stepping stone		
t_C	mean	se	mean	se	m_B/m	mean	se
0	0.114	0.006	0.114	0.006	0.002	0.091	0.014
1	0.996	0.008	0.655	0.024	0.010	0.310	0.027
2	0.999	0.009	0.746	0.022	0.020	0.462	0.032
3	0.999	0.007	0.780	0.024	0.100	0.828	0.025
4	1.000	0.008	0.800	0.020	0.200	0.913	0.020
5	1.000	0.006	0.814	0.017	1.000	1.001	0.010

Table S4: FST ratio.

References

Rousset, F., 1997. Genetic differentiation and estimation of gene flow from F-statistics under isolation by distance. Genetics 145:1219–1228.

Sedghifar, A., Y. Brandvain, P. Ralph, and G. Coop, 2015. The spatial mixing of genomes in secondary contact zones. Genetics 201:243–261.
